# Larval therapy vs conventional silver dressings for full-thickness burns: a randomized controlled trial

**DOI:** 10.1186/s12916-023-03063-7

**Published:** 2023-09-19

**Authors:** Jasem Gaffari, Kamran Akbarzadeh, Mozhgan Baniardalani, Reza Hosseini, Safdar Masoumi, Zahra Sadat Amiri, Razieh Shabani Kordshouli, Javad Rafinejad, Mostafa Dahmardehei

**Affiliations:** 1 Department of Medical Entomology and Vector Control, School of Public Health, Tehran University of Medical Sciences, Tehran, Iran; 2 Cancer Research Center, Shahid Beheshti University of Medical Sciences, Tehran, Iran; 3 Department of Biostatistics, Faculty of Medical Sciences, Tarbiat Modarres University, Tehran, Iran; 4 Burn Research Center, Iran University of Medical Sciences, Tehran, Iran

**Keywords:** Larvae, Maggot therapy, Silver dressings, Burn, Infection

## Abstract

**Background:**

This is the first clinical trial to investigate the effectiveness of maggot debridement therapy (MDT) for full-thickness burn injuries in comparison to conventional silver dressings.

**Methods:**

Thirty-one cases with full-thickness (grade III based on ICD-10 classifications version 2019) burns were assigned into larval therapy (15 cases) and conventional treatment (16 cases) groups. Participants in the MDT group have received loose larvae on days 0, 2, 4, and 6, while controls received a conventional regimen comprised of sharp debridement, silver sulfadiazine, antibiotic therapy, and offloading every day. The primary and secondary outcomes were defined as the time to debridement (from admission to skin autograft) and time to healing (from admission to complete healing post-skin autograft). Patients in two groups were also compared in terms of necrosis resolution, granulation, and granulation/necrosis (g/n) ratio during study time periods.

**Results:**

Participants who received larvae had significantly decreased necrosis on days 2 (*p* = 0.028) and 4 (*p* = 0.023) compared to those who received control treatment. Significant differences (*p* < 0.001) were also observed for granulation between the two groups in favor of MDT and the fold changes of g/n in the larvae group were 5, 15, and 13 times higher than that for the conventional regimen on days 2, 4, and 6 of treatment, respectively. Strikingly, a subgroup analysis of high necrotic burns (necrosis > 50%) revealed a significant improvement (*p* < 0.001) for MDT compared to the control treatment. There were also significant differences (*p* < 0.001) for the time to debridement and time to healing between the two groups. However, bacterial contamination did not show significant changes between the two treatment regimens.

**Conclusions:**

Our findings revealed that MDT has a favorable superiority over conventional regimen for the treatment of grade-III burns, and thus further clinical trials with larger sample size are warranted to confirm these results.

**Supplementary Information:**

The online version contains supplementary material available at 10.1186/s12916-023-03063-7.

## Background

Burns are considered as one of the commonly occurring traumatic injuries worldwide, leading to life-long disabilities and adverse health, social, and economic consequences [1]. Statistics show an incidence of 11 million burns of all types throughout a year globally, most of which (~ 90%) occurring in low- and middle-income countries [1–3]. It is estimated that 180,000 deaths occur annually due to severe burns all over the world, which might be caused by exposure to heat or cold, chemical agents, radiation, electric sources, etc. [4, 5]. The tissue destruction due to burn injuries can also be at different grades, since the exposure sources/levels might be different [6]. For instance, exposure to flame can lead to deep burn injuries, whereas chemical exposure might lead to highly necrotic wounds, which can therefore affect the treatment modalities [1, 6, 7].

Based on their severity, burn injuries are classified into different grades, where burns that entirely affect the uppermost skin layer are classified as grade I, while deep partial-thickness burns are considered as grade II [1]. On the other hand, full-thickness burns, affecting full dermis, and burns that damage deeper tissues, including muscles and bones, are categorized into grades III. The two former burn injuries (grades I and II) may not need surgical interventions and would be managed with topical antimicrobial dressings [1]. However, severe burns (grades III) involving > 10 of the total body surface area (TBSA) need intensive care and surgery [1, 8]. Although significant progresses have been made over the years in managing severe burn injuries (grade III), it is still a vivid research area to enhance treatment options [8, 9]. The conventional treatment for severe burns comprises non-surgical interventions (including silver dressings, ointments, and creams) along with surgical excision of necrotic areas (debriding) [9]. However, because of the risk of burn wound sepsis, cost-effectiveness issues, and less efficacy of conventional therapy, numerous attempts are being made to devise novel treatment approaches.

In recent years, biological therapies have attracted great attention in treating burn wounds and are widely studied for potential clinical use [10, 11]. Dressing with topical agents (e.g., plant extracts or derivatives) as well as stem cells has resulted in promising outcomes [12, 13]. However, due to some defects or concerns, the clinical practice of these derivatives or cellular components for burn healing is still a matter of debate [13, 14]. Contrary, the use of *Lucilia Sericata* maggots/larvae (maggot debridement therapy, MDT) for the management of several chronic wounds, including diabetic ulcers and bed sores were shown to be beneficial in clinical trials [15–17]. Based on the literature, larval therapy can debride wounds more swiftly compared to conventional treatments [18]. Mechanistically, maggots were shown to stimulate the healing process (angiogenesis, proliferation, and remodeling), debriding necrotic tissue, and eradicating bacterial infections, especially those methicillin-resistant *Staphylococcus aureus* species [18, 19]. However, the common side effects associated with MDT are pain at the application site as well as phobia [18, 20]. Although the painful sensation due to maggots can be soothed by oral analgesics, but maggot phobia patients may be more treatment-compliant or request the early termination of the MDT.

In addition to their beneficial effects in treating diabetic ulcers and bed sores, there are several case reports demonstrating that MDT can also improve the healing process in burn injuries [21–24]. Of note, maggots were shown to reduce necrosis and the microbial loads of burns and increase granulation tissue that positively affects patients’ outcomes and decreases the needs for antimicrobial therapy and the incidence of secondary infection induced-sepsis, as a prominent complaint in severe burns [24, 25]. However, whether MDT can favor severe burn injuries (grade III) has not been investigated yet. On this basis, in the present study, we aimed to study the beneficial effects of MDT on burn injuries in comparison to conventional silver dressing regimen. To the best of our knowledge, this is the first clinical trial examining the therapeutic effects of maggots in treating grade-III burn wounds vs the conventional regimen. Our findings are a proof of concept that MDT could potentially be considered a more effective and available therapy used in clinical settings for treating high-grade necrotic burns. However, further randomized clinical trials with larger sample size would shed more light on the efficacy of MDT in treating burn wounds.

## Methods

### Patients and study design

**Fig. 1:**
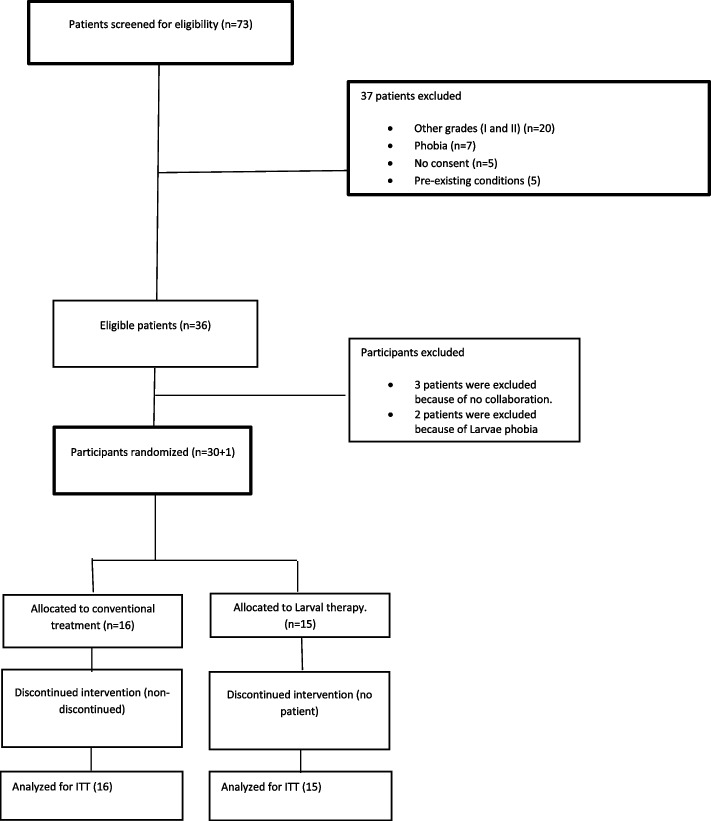
Flow of participants enrolled in this study. The present study was conducted based on CONSORT guidelines for reporting clinical trials

This open randomized controlled trial was carried out at Shahid Motahari Burns Hospital, Tehran, Iran, from November 2018 to May 2020. Participants were 31 cases with at least one full-thickness (grade III based on ICD-10 classifications) burn that referred to Shahid Motahari Burns Hospital related to Iran University of Medical Sciences, Tehran, Iran. The flow of participants is shown in Fig. 1. The patients were randomly assigned to either the larvae or the conventional groups by block randomization. A block size of four was used for randomization, and the sequences were calculated by Random allocation software v. 2.0. The research coordinator had exclusive access to the numbered, sealed, opaque envelopes that indicated each patient’s randomized treatment assignment. As an open-label study, the patients, care providers, and outcomes assessors were all informed of the treatment assignments. Indeed, the nature of the maggot therapy would not allow the study to be done in a blinded manner. The sample size was calculated based on the time to debridement (primary outcome) in the larvae group versus the conventional group. We assumed the anticipated effect size of *f* = 1.05 (mean (SD) = 10 (3) and 13.7 (4.3) days for the larvae group and conventional group, respectively), type I error of 0.05, and test power of 90% based on Muangman P et al.’s study [26]. A total sample size of *n* = 34 was calculated with G*Power 3.1 (University of Kiel, Germany) using two-sample independent *t*-test. However, after all participants completed at least 6 days of follow-up, an error was found in the original sample size calculation, and the planned 31 participants actually provided a power of 0.88 to detect a mean difference of 3.7 days. The statistic details of the sample size calculation are provided in Additional files 1 and 3.

All the cases in the larvae group were informed about the methodology and study purposes and enrolled as volunteers, with a signed consent letter. Patients who had phobia or other serious per-exiting conditions including the presence of gangrene, severe pain, refractory to treatment, and immunocompromised patients (e.g., HIV/AIDS) and those who have been receiving steroids and anti-coagulants that might affect the results were excluded from the study. Furthermore, probable bleeding because of larvae therapy and causing slough burns were also considered as exclusion criteria. All the procedures were in accordance with the Helsinki Declaration regarding human research and were reviewed and approved by the Medical Ethics Board of Tehran University of Medical Sciences, Tehran, Iran (with code no. IR.TUMS.VCR.REC.1396.4691) [27]. This study has also been registered in the Iranian registry of clinical trials and received a clinical trial code (IRCT ID: IRCT20170531034272N2).

### Interventions

As mentioned earlier, eligible participants were those who had full-thickness (grade-III based on ICD-10 classifications version 2019) burns based on the pathological examinations and expert opinions irrespective of the size and necrosis rate of wounds. The larvae group and controls were 15 and 16 patients with full-thickness (grade-III) burns, respectively. In the intervention group, each patient has received loose larvae 3–4 times (5–10 larvae/cm^2^ of burn area as described elsewhere for diabetic ulcers), with 2 days’ interval (day 0, day 2, day 4, and day 6) [28, 29]. On the other hand, the control group received the conventional regimen for full-thickness burns (sharp debridement, silver dressings, antibiotic therapy, offloading). Of note, in the conventional treatment group, burn dressings were replaced every other day by expert nurses (3 times/week).

### Data collection and outcome measurements

Demographics of patients including age, sex, and baseline data on burn mechanisms (exposure source), site of burning, and necrotic and granulation tissues were collected at admission to the hospital and summarized in Table 1. Furthermore, related data on burns after the application of treatments was measured every 48 h (during the replacement of dressings) under the observation of doctors specialized in dermatology and burning. To examine the therapeutic effects of larvae in comparison to conventional treatment, the surface areas of granulation and necrotic tissues were photographed at days 0, 2, 4, and 6 and analyzed by Image J Software. In detail, as can be seen in the images, the wound size was also measured using a ruler. The granulation and necrosis percentage of each wound during the treatment course was observed by 3 independent expert dermatologists (examiners) and a mean value was calculated for these measurements. At the same time, the Image J Software was employed to measure the granulation and necrosis based on the observations of 3 independent expert examiners and then a mean value was calculated. Afterward, the interexaminer reliability study was done as described elsewhere [30]. In this regard, the standard deviation (SD) was obtained for the calculated mean values and then Spearman’s correlation coefficient was measured for these values. In the next, the error term (denominator) was calculated using independent *t*-test. Finally, the reliability coefficient was calculated using the following formula:

$$R=\frac{{\sigma }_{T}^{2}}{{\sigma }_{T}^{2}+\frac{1}{k}\sum {\rho }_{j}^{2}+{\sigma }_{e}^{2}}$$

where σ^2^_*T*_ is the SD of calculated mean values, *ρ* is the Spearman’s correlation coefficient, σ^2^_e_ is the error term between mean values, and *K* is the number of examiners. The results were assumed reliable if the R value was above 0.8.

**Table 1:** Demographics and clinical data of burn patients in both treatment arms

**Variable**	**Total** ***N* = 31**	**Larvae** ***n* = 15**	**Conventional therapy** ***n* = 16**	*P value*
**Men, *n* (%)**	31 (100)	15 (100)	16 (100)	-
**Age, yrs**				
20–40	10	4	6	0.76
40–60	14	6	8	
> 60	7	5	2	
Mean ± SD	48.4 ± 18.6	45.3 ± 22.8	50.3 ± 15.8	
**Mechanism,* n* (%)**				
Boiling water	7	3	4	0.83
Chemical	6	3	3	
Electrical	14	7	7	
Radiation	1	1	0	
Flame	3	1	2	
**Burning site, *n* (%)**				
Hand	18	9	9	0.86
Foot	10	5	5	
Other	3	1	2	
**Type infection**				
*Staphylococcus*	11	7	4	0.15
*Pseudomonas*	13	7	6	
Both	4	0	4	
Others	3	1	2	
**Burn degree (grade III based on ICD-10 classifications)**	31 (100)	15 (100)	16 (100)	-
**Size wound (cm**^**2**^**)**				
5–100	16	7	9	0.59
> 100	15	8	7	
**Necrosis (%)**				
$\le$ 50	13	7	6	0.60
$>$ 50	18	8	10	

Of special note, to further minimize interpretation bias, the results were also adjusted to the would size.

**Fig. 2:**
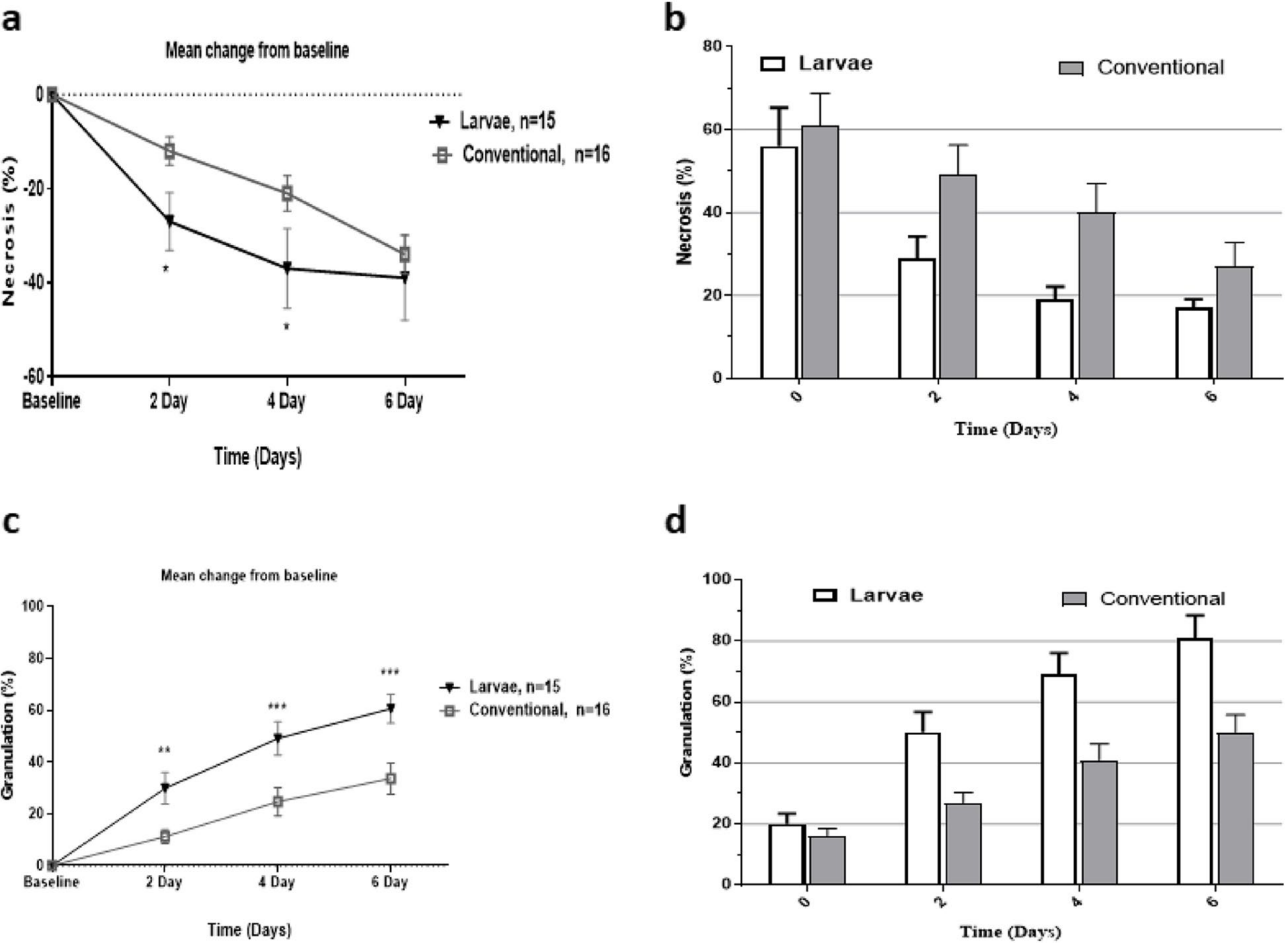
Necrosis and granulation changes between and within groups received larvae or conventional therapy. Necrosis was significantly decreased in patients who received larvae on days 2 and 4 compared to baseline and conventional treatment groups. The larvae group had significantly improved granulation throughout the interventional duration. Error bars represent the standard deviation for the bar charts and the standard error of the mean for scatter plots. * shows statistical differences between groups, *P < 0.05, **P < 0.01, ***P < 0.001

The primary outcome in this study was considered as time to debridement that was defined as the time in which burns were cosmetically clean and participants were ready to receive transplantation (skin autograft). Digital photographs were taken during every 2 days, when old larvae dressings were replaced with new ones, and are presented for each patient in Additional file 2, with a representation for each group in Fig. 2. The secondary outcome was considered as the time to healing that was defined as the duration from the admission to the complete healing post-skin autograft based on the experts’ opinion. Of note, the complete healing is described as the wound closure, no pain, fever, discoloration, redness, swelling, and a breakdown of tissue at the grafted site.

### Rearing fly eggs and preparing larvae

We used *Lucilia sericata* larvae for the maggot therapy in this study, which is continuously reared in the fly insectary of Tehran University of Medical Sciences from 2013. As described elsewhere, *L. sericata* eggs were harvested from adult fly cages, disinfected chemically, and placed in sterile containers for therapeutic applications [29]. For disinfection purposes, *L. sericata* eggs were washed with distilled water after harvesting and then were submerged in the chlorhexidine 5% and EtOH 70% for 8 and 3 min, respectively. Afterward, the eggs were washed again with sterile distilled water and placed in flasks containing sterile blood agar media and incubated for at 28–30 °C. After 24 h, the disinfected larvae were ready for dressing applications.

### Dressing and debridement

Before applying the maggots, the wounds were cleansed by saline, and then zinc oxide was used on the healthy skin around the wounds (to protect the skin from irritation or maceration from the proteolytic wound drainage and maggot secretions). Dressings were prepared by cutting an opening in the sterile gauze as the shape of the wound. The sterile gauze was then employed over the barrier-protected periwound skin. This was done to restrict the larvae to the wound bed and to create a space for their activity. In detail, 5 to 10 maggots/1cm^2^ were applied directly onto the wound surface at 48-h intervals. A sterile soft mesh net of polyvinyl alcohol was also used for covering the maggots on the wound with a purpose to supply enough oxygen. The drainage of burn wounds was also absorbed by replacing 1 to 3 sterile gauze pads over the wound bed. Furthermore, the dressings were secured with soft roll bandage and were removed every two 2 days for examining debridement, necrosis and granulation status, and bacterial contamination and recorded in patient’s clinical cases. It should be mentioned that physiological serum was employed for removing the remaining larvae debris from the burning site.

### Bacterial sampling

Since bacterial contamination is a common feature of grade-III burns, therefore we investigated whether MDT could affect bacterial load of burn wounds [31]. For this purpose, the swab technique was employed to collect samples from burns for bacterial cultures before each maggot application. Briefly, burning sites were washed with normal saline and the periwound area was carefully sterilized with 70% alcohol. Then, sterile swabs were used to collect samples from burning area and transferred into transport media (Trypticase Soy Broth). The sample containers were labeled with sampling date, patients’ name, and the intervention time and the collected samples transferred to microbiology laboratory for further cultures to determine the bacterial species and contamination level. Of note, synchronized sampling was also done from the wounds in the control treatment group. Afterward, the transport media containing the bacterial samples were cultured in blood agar (5% sheep blood) and MacConkey media for gram-negative bacteria and were incubated for 24–48 h at 37 °C. Smears were provided from suspicious colonies and were cultured in TSA to obtain pure colonies. After obtaining individual colonies, smears were prepared for optical microscopy and examined in terms of their gram-negative or -positive nature, and differentiation tests were carried out to determine *Pseudomonas aeruginosa* and *Staphylococcus aureus.*

### Statistical analysis

All the analyses were performed by Prism 8.1 and STATA version 14 softwares. The normal distribution of data was checked using Kolmogorov–Smirnov test. Significant levels were considered as confidence interval of 95% (CI 95%) and *P* value < 0.05 and the trial were analyzed using the principle of intention to treat (ITT). Demographics of patients at baseline were shown with mean ± SD and categorical variables were presented as frequency and percent. The time to debridement (primary outcome) and time to healing (secondary outcome) between the two groups were compared using log-rank test (Kaplan–Meier curves). The interventional effects on necrosis, granulation, and granulation/necrosis ratio between and within groups in different time points were analyzed using generalized estimating equations (GEE) model for longitudinal data. In GEE model interventional effects were adjusted for baseline characteristics (burn size). The effect size was presented by Cohen’s *d* (95% CI). Data on bacterial contamination was descriptively reported as the number of burn samples (cases) that have been detected for contamination with *Staphylococcus aureus* and *Pseudomonas aeruginosa* species and analyzed using Pearson’s chi-squared test. Of note, bacterial contamination was analyzed from day 0 to the end of the debridement phase (day 6).

## Results

Thirty-one cases with full-thickness burn wounds were included in this study. Fifteen and 16 patients were assigned to larvae (MDT) and conventional treatment (silver sulfadiazine, etc.) groups, respectively, and their baseline characteristics are summarized in Table 1.

All the patients enrolled in this study were males. The majority of the cases aged 40-60 years, and the mean ± SD of participants’ age was 48.4 ± 18.6. The burn wounds were due to boiling water (7 cases), chemical exposure (6 cases), electrical origin (14 cases), radiation exposure (1 case), and direct contact to flame (3 cases). Most of the wounds were located in hands/shoulders (18 cases) and legs/foots (10 cases). Initial sampling of burn sites showed that 11 and 13 burns were infected with *Staphylococcus* and *Pseudomonas aeruginosa*, respectively, while 4 burns were infected with both strains. All the burns were considered full-thickness (grade III) based on the ICD-10 classifications version 2019. The wounds were categorized into 5–100 cm^2^ (16 burns) and > 100 cm^2^ (15 burns). Seven and 9 patients in the larvae and conventional groups had wound sizes of 5–100 cm^2^, respectively, while 8 and 7 cases in the larvae and conventional groups had burn sizes of > 100 cm^2^, respectively. Moreover, based on the initial necrosis at the time of admission to hospital, 13 cases had necrosis less than 50%, while 18 cases had burns with > 50% necrosis according to the experts’ opinion. As it can be observed in Table 1, there are no significant differences in the baseline characteristics of the participants assigned into larval therapy and conventional treatment groups in terms of age, mechanism of burn induction, burn sites and grade, type of infection, wound size, and necrosis percentage.

As indicated in Table 2 and Fig. 2, within and between-group differences in terms of necrosis resolution, granulation, and granulation/necrosis ratio on days 2, 4, and 6 compared to the baseline value were calculated. It should be noted that the baseline value for necrosis in the primary wound (day 0) was considered as the 100%, while the granulation was considered 0% at day 0. Then, the effects of treatment on the percentage reduction of necrosis and granulation increase were measured compared to that baseline status. To minimize the bias, the findings were also adjusted for the wound size (cm^2^). As shown in Fig. 2, both in larval therapy and conventional groups, the mean percentage of necrosis is reduced, while granulation percentage is increased from day 0 to day 6; however, these changes are more remarkable for larval therapy. As summarized in Table 2, the within-group results show that the mean change of necrosis reduction and granulation increase from baseline (day 0) to days 2, 4, and 6 were statistically significant in both interventions. The granulation/necrosis (g/n) was found to be significantly changed for the larval therapy during the treatment course. However, the g/n ratio for the conventional treatment group was only significant at days 4 and 6 of the study period. Interestingly, between-group analysis revealed that the patients who received larvae had higher necrosis reduction at days 2 (*p* = 0.028) and 4 (*p* = 0.023) compared to those patients treated with conventional regimen. The granulation was also significantly increased in larval therapy patients compared to the patients who received silver dressings. In addition, a between-group comparison of the g/n ratio showed a significant increase in favor of larval therapy on days 4 and 6 of the study course.

**Table 2:** Mean difference within-group and between-group during treatment intervals (ITT)

	**Test of within-group effects (mean change from baseline)**		**Test of between-group effects (mean change control group)**			
	**Larvae, *n* = 15**	**Conventional, *n* = 16**	**Larvae vs conventional**			
**Outcomes**	**Mean [95% CI]**	**Mean [95% CI]**	**MD**	**95% CI**	***P *v**	**Cohen’s *d* [95% CI]**
**Necrosis (%)**						
Baseline to day 2	− 0.27 [− 0.38, − 0.15]^***^	− 0.12 [− 0.18, − 0.16]^***^	** − 0.14**	**[− 0.28, − 0.01]**	**0.028**	0.91 [0.54, 1.27]
Baseline to day 4	− 0.37 [− 0.49, − 0.25]^***^	− 0.21 [− 0.28, − 0.15]^***^	** − 0.15**	**[− 0.28, 0.02]**	**0.023**	0.73 [0.37, 1.08]
Baseline to day 6	− 0.39 [− 0.51, − 0.27]^***^	− 0.34 [− 0.40, − 0.28]^***^	− 0.05	[− 0.18, 0.08]	0.45	0.38 [− 0.12, 0.88]
**Granulation (%)**						
Baseline to day 2	0.30 [0.21, 0.39]^***^	0.11 [0.03, 0.19]^***^	**0.18**	**[0.06, 0.31]**	**0.003**	1.05 [0.68, 1.42]
Baseline to day 4	0.49 [0.39, 0.59]^***^	0.24 [0.16, 0.33]^***^	**0.24**	**[0.11, 0.37]**	** < 0.001**	1.06 [0.69, 1.43]
Baseline to day 6	0.60 [0.51, 0.70]^***^	0.34 [0.25, 0.43]^***^	**0.26**	**[0.13, 0.38]**	** < 0.001**	1.20 [− 0.12, 0.88]
**Granulation/necrosis**						
Baseline to day 2	5.01 [0.08, 9.94]^*^	0.23 [− 0.47, 0.94]	4.77	[− 0.22, 9.77]	0.06	0.48 [− 0.08, 1.04]
Baseline to day 4	15.5 [10.6, 20.5]^***^	0.81 [0.10, 1.52]^*^	**14.7**	**[9.74, 19.73]**	** < 0.001**	1.37 [0.58, 2.13]
Baseline to day 6	13.7 [8.8, 18.6]^***^	1.43 [0.70, 2.16]^***^	**12.32**	**[7.28, 17.37]**	** < 0.001**	2.72 [1.72, 3.70]

^*^P < 0.05

^***^P < 0.001

^a^Adjusted generalized estimating equations model after controlling the baseline outcome, wound size (cm^2^)

**Fig. 3:**
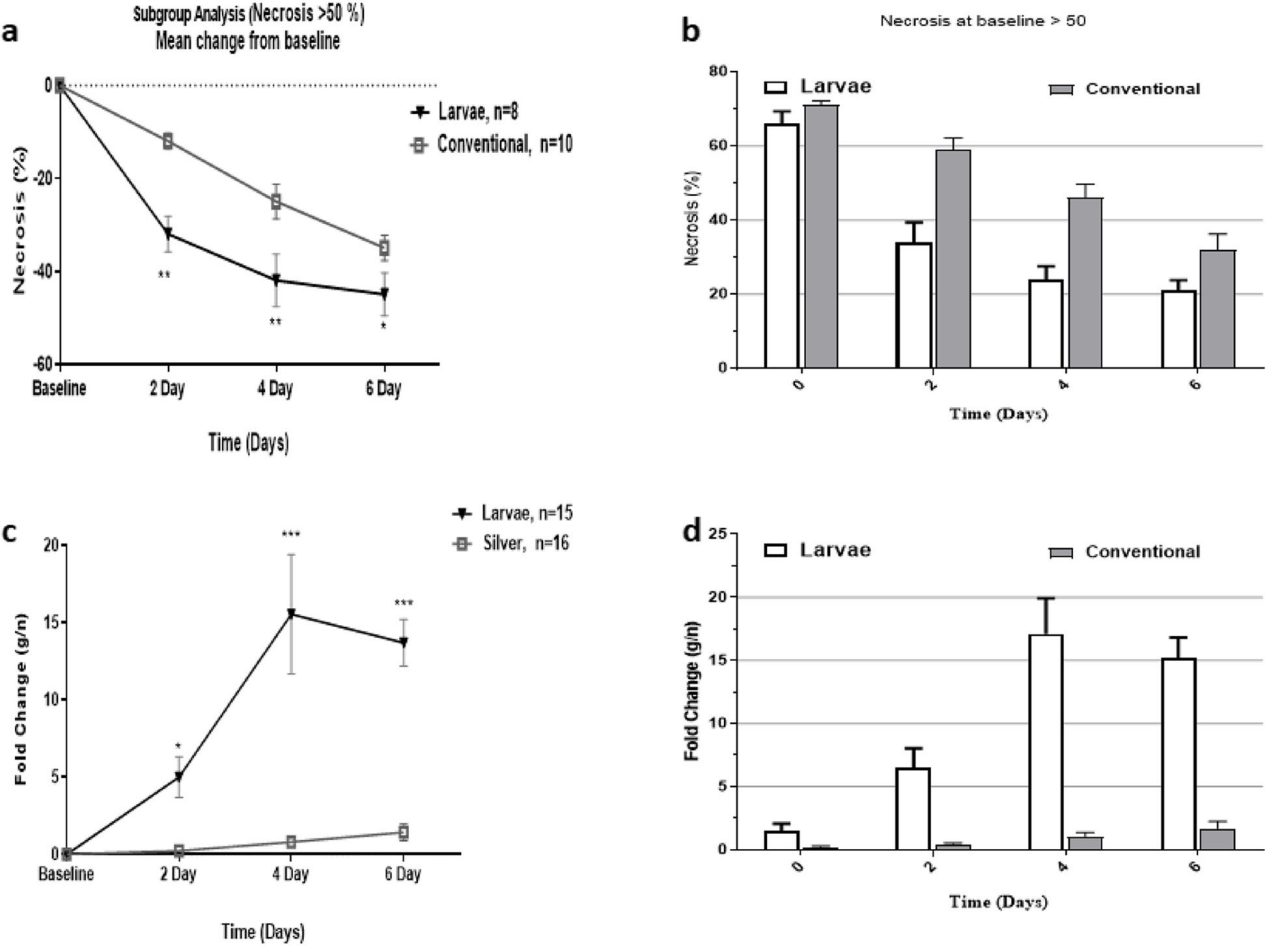
Subgroup analysis for necrosis > 50%. Larval therapy was able to remarkably decrease necrosis in high necrotic burns (necrosis > 50%) in comparison to conventional treatment. The fold changes of g/n in the larvae group exhibited significant differences compared to the baseline and conventional regimen in the same time points. * shows statistical differences between groups, *P < 0.05, **P < 0.01, ***P < 0.001

We also performed subgroup analysis to determine the efficacy of larvae debridement therapy (MDT) in the treatment of high necrotic burns (> 50%) comparing to conventional treatment. As shown in Fig. 3, the results of subgroup analysis revealed that larvae intervention significantly improved high necrotic burns on days 2, 4, and 6 compared to the conventional silver sulfadiazine treatment. Of particular note, the fold change of g/n ratio in the larvae group was 5, 15, and 13 times higher than that obtained for the conventional regimen on days 2, 4, and 6, respectively.

**Fig. 4:**
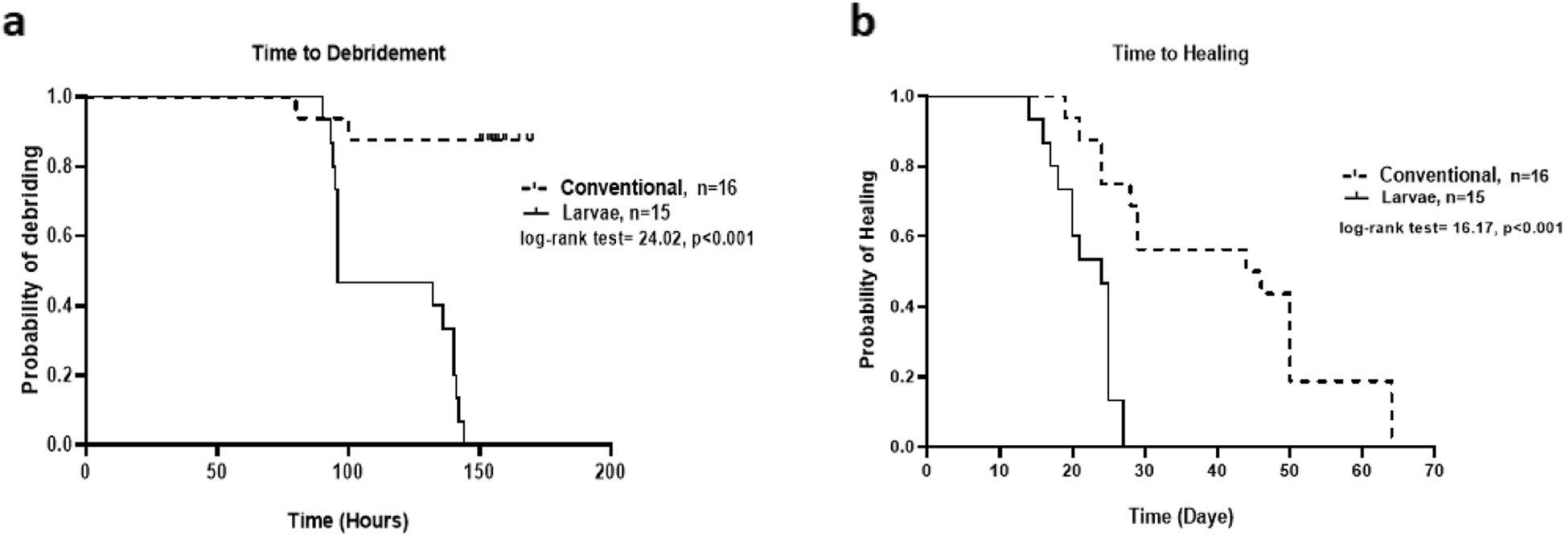
Larval therapy significantly decreased **a** time to debridement (primary outcome) and **b** time to healing in grade-III burn patients versus conventional treatment

**Fig. 5:**
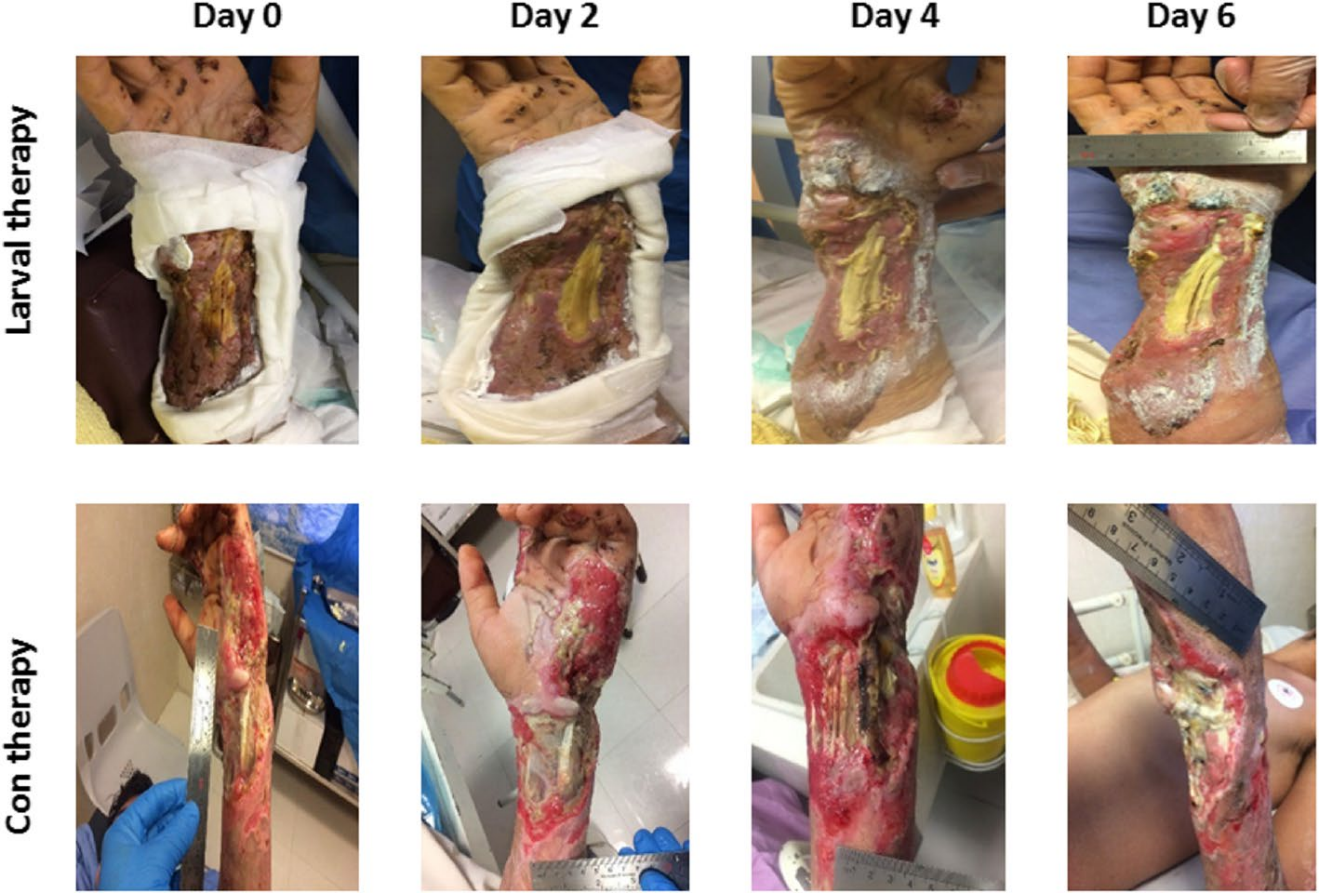
Graphs representatively show the efficacy of larval therapy in debriding grade-III burns compared to conventional treatment within the study timeline. Con, conventional

As previously described, we have evaluated the time-to-debridement and time-to-healing as the primary and late outcome measures. We observed that the time-to-debridement was significantly differed between larvae and routine treatment groups. The median time-to-debridement in the larvae group was 96 h (95% confidence interval 95 to 140) and in the conventional treatment group was 156.5 h (95% confidence interval 152 to 165). These findings are illustrated in Fig. 4a using Kaplan–Meier survival analysis. As summarized in Table 3, at the end of day 6, all of the burns in the larvae group were entirely debrided; however, only 2 of 16 cases in the silver sulfadiazine treatment group had full debridement. These results indicate that the time-to-debridement in the larvae group was significantly shorter than that observed for conventional regimen. Figure 5 is a representative graph showing the debriding efficacy of larval therapy versus the conventional regimen from day 0 to day 6. Similar graphs for all the patients who received either larval therapy or conventional regimen are also available in Additional file 2.

**Table 3:** Primary (time-to-debridement) and secondary (time-to-healing) outcomes in patients who received larval therapy or conventional treatment

**Outcomes**	**No. of cases**	**Time [median (IQR)]**	**No. of event**
**Debridement**			
Total	31	142 (96–157)	17
Larvae	15	96 (95–140)	15
Conventional	16	156.5 (152–165)	2
**Healing**			
Total	31	25 (21–46)	31
Larvae	15	24 (18–25)	15
Conventional	16	45 (26–50)	16

As mentioned earlier, the time-to-complete healing of burn wounds after surgery and skin autograft was considered a secondary (late) outcome. Based on the results from Fig. 4b, it is obvious that the time-to-healing is significantly different between the two groups of interventions. The median time-to-healing was significantly (*p* < 0.001) shorter in the larvae group (24 days, 95% confidence interval 18 to 25) compared to those who received conventional treatment (45 days, 95 confidence interval 26 to 50). It should be mentioned that all the participants in both groups completed the remedy and no patient failed the treatment.

**Fig. 6:**
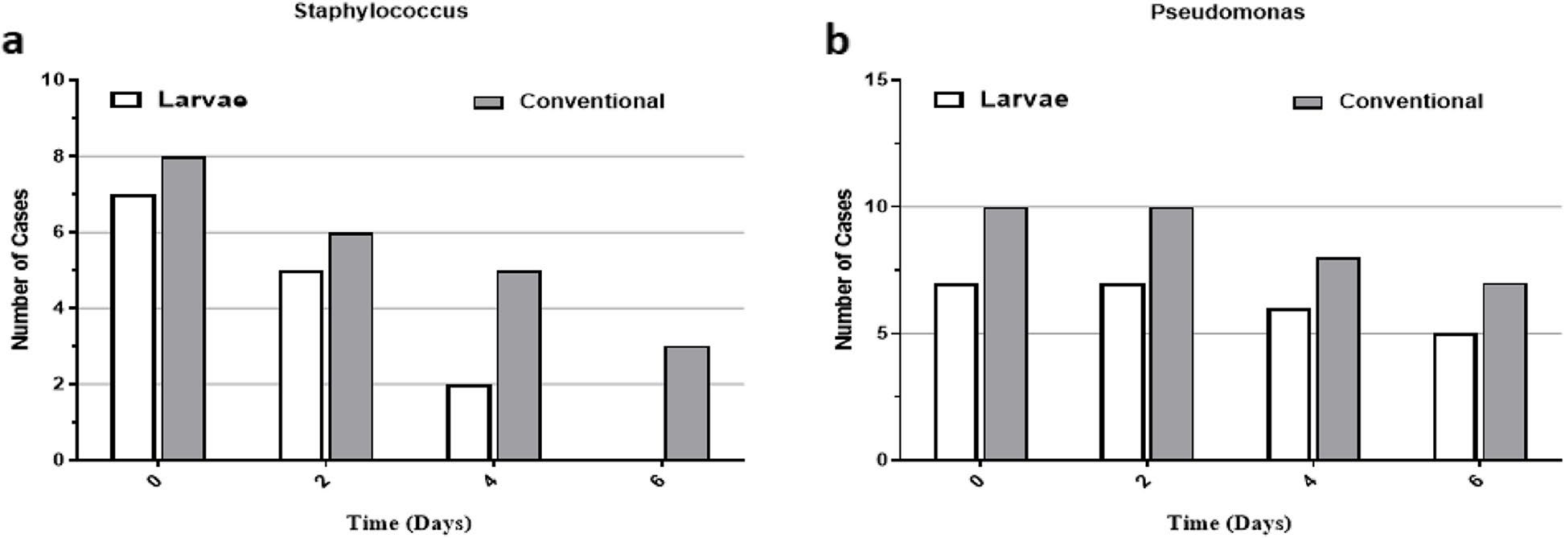
Trends of bacterial load in burn injuries of patients treated with larvae compared to conventional therapy. Treatment with larvae had superior microbicidal effects on both **a ***Staphylococcus* and **b ***Pseudomonas* species relative to conventional treatment

Since previous reports have shown an antimicrobial effect for *L. sericata* larvae as part of its healing benefits, we have also examined the effect of both interventions on the resolution of infections. Based on the findings summarized in Table 4 and Fig. 6, no significant differences were observed between the two interventions. However, within-group assays showed that both modalities could significantly eradicate *Staphylococcus* infection during the interventional timeline, whereas no statistical changes were found for burns with *Pseudomonas aeruginosa* infection.

**Table 4:** Differences (between and within groups) in bacterial cultures of burn patients who received larvae or conventional therapy

**Contamination**	**Larvae**	**Silver**	*P* value Between group
***Staphylococcus***			
Baseline (day 0)	7	8	0.51
Day 2	5	6	
Day 4	2	5	
Day 6	0	3	
*P* within group	0.016	0.06	
***Pseudomonas***			
Baseline (day 0)	7	10	0.84
Day 2	7	10	
Day 4	6	8	
Day 6	5	7	
*P* within group	0.50	0.25

## Discussion

This study is the first clinical trial to compares the therapeutic potency of maggot debridement therapy (MDT) for full-thickness (grade-III) burn injuries versus conventional treatment regimen. We found that patients who received maggots had significantly improved granulation and decreased necrosis within the same timeline compared to the conventional treatment group. As it is summarized in Table 2 and Fig. 2, maggot intervention would be able to significantly decrease necrosis compared to the control group on days 2 and 4 of treatment. The differences between maggot therapy and conventional groups in terms of necrosis on day 6 of treatment were borderline significant. On the other hand, patients who received maggots had significantly better granulation on days 2, 4, and 6 compared to the baseline and conventional treatment group. These findings indicate that treatment with maggots could result in better outcome within the same time of treatment compared to conventional regimen. Although there is no clinical trial in the literature examining the beneficial effects of larval therapy in burn wounds, but from the previous experiences on the diabetic ulcers and bed sores it can be explained that debriding wound area might be most responsible for our observations [19]. Indeed, *L. sericata* larvae are proven to perform microdebridement in the wound area and cleanse it from the necrotic tissue [19].

On the other hand, these maggots can also scavenge microbial infection from the affected surface. In addition, *L. sericata* larvae were also shown to secrete several antimicrobial agents including defensin and cathelicidin that further enable them to eradicate infection [19]. Together, through these mechanisms, the use of MDT for burn injuries can be justified [19]. The beneficial effects of MDT on the increase of granulation tissue can also be explained by the similar mechanisms [32]. Previous studies have shown that regardless of microdebridement and cleansing infection, the secretome of *L. sericata* larvae was shown to induce cellular proliferation and tissue remodeling via increasing the transforming growth factor (TGF)-β gene expression levels, a critical cytokine that is involved in the tissue healing process [32, 33]. This subsequently induces granulation tissue that is characterized histologically by the presence of fibroblasts, keratinocytes, endothelial cells, new thin-walled capillaries, and inflammatory cell infiltration of the extracellular matrix [33].

To minimize the bias, we also performed a subgroup analysis for patients who had > 50 necrosis at the baseline, to investigate whether MDT or conventional treatment could be more potent in high necrosis burns. Of particular note, we found that MDT had a significant superiority to conventional regimen in treating high necrotic wounds. We defined a novel index of granulation/necrosis (g/n) to elucidate the efficacy of MDT in comparison to routine treatment for grade-III burns. As shown in Fig. 3, the fold changes of g/n in the MDT group were 5, 15, and 13 times higher than those found in the conventional treatment group. Indeed, such a finding shows that treatment with MDT could fasten healing process and may result in better outcomes. This idea is evident, as a significant difference was observed in the log-rank of the time to debridement between the MDT group and conventional group. Moreover, we have also examined whether there is a difference in the time to healing (defined as admission to post-transplantation healing) between intervention groups. Likewise, we observed a significant improvement in time to healing for the MDT group (Fig. 4). Collectively, these findings indicate that larvae are more effective debriding agents compared to conventional regimen and are directly associated with favorable outcomes. Similar findings were also reported in the literature. For example, in a previous study, Dumville et al. have examined the clinical effectiveness of larval therapy with a standard debridement technique (hydrogel) for sloughy or necrotic leg ulcers [15]. Their findings revealed that larval therapy can significantly reduce time-to-debridement compared to the standard technique. The authors have assumed that this can be due to the higher debriding capability of the larval therapy compared to hydrogel [15]. Since high-grade burn injuries are mainly comprised of necrotic tissue and *L. sericata* larvae which mainly act as debriding agent, MDT can be more effective in this context compared to slough diabetic wounds.

Bacterial contamination of high-grade burn injuries is also another matter of importance which affect the treatment length, as well as the final outcome [34]. Contamination with *Pseudomonas aeruginosa* and *Staphylococcus aureus* are two common types of co-infections that are managed by the administration of antibacterial agents [34, 35]. To this end, we have examined the beneficial effects of MDT on bacterial contamination of burn wounds compared to conventional therapy. Although, we found no significant differences between the two groups of treatments, but MDT had more favorable effects in removing bacteria from burn injuries (Table 4, Fig. 6). These results indicate that MDT has the potential bactericidal effects which can reduce the need for high-dose antimicrobial agents. Similar results have also been reported by previous studies [25]. Although it is not well-known, but similar to our result previous studies have also shown that MDT is more useful in eradicating *Staphylococcus aureus* rather than *Pseudomonas aeruginosa *[36–39]. Also, it has been shown that *L. sericata* larvae could affect the healing process at least by dissolving bacterial biofilm covering the wound surface [36–39]. Furthermore, larval therapy also prevents the growth of new biofilm through which promotes granulation tissue.

## Strengths and limitations

Although several case reports have been reported, however, this is the first trial that has investigated the therapeutic potential of Larvae for burn injuries in regard to conventional therapeutic regimen. The findings of this study is a proof of concept that in addition to their benefits for diabetic leg ulcers, larvae can also improve the healing process in burn wounds and have the potential to be used as an effective alternative or complementary for high-grade burns [15, 17]. More interestingly, our results indicated that larval therapy could be an interesting option in treating high necrotic burns, which marks it as an ideal choice for clinical settings. However, future randomized controlled clinical trials with sufficient numbers of eligible patients are also warranted to further elucidate the beneficial effects of larval therapy alone or in combination with existing therapies for treating burn wounds.

## Conclusions

Our results showed that patients with grade-III burns who received larval therapy had remarkably improved granulation and decreased necrosis within 6 days of intervention compared to baseline and conventional regimen group. Moreover, treatment with Larvae had a significant superiority in high necrotic (> 50%) burns over conventional treatment. Additionally, maggot therapy was able to remove microbial contamination from burn comparable to that seen in the conventional group who have been given antibacterial agents. In summary, the findings of this study provide the basis for the application of larvae in treating grade-III burn wounds. However, further randomized clinical trials with larger sample size should be done to shed light on the different aspects of the MDT in treating grade-III burn wounds, as well as grades I and  II.

### Supplementary Information

**Additional file 1.** Sample size calculation.

**Additional file 2. **Graphic presentations of participants received Larvae (cases 1-15) or conventional treatment (cases 16-31) from day 0 to day 6.

**Additional file 3. **(Study protocol).

## Data Availability

The data that support the findings of this study would be available upon a reasonable request and editorial office permission.

## Abbreviations

AIDS: Acquired immunodeficiency syndrome
EtOH: Ethanol
GEE: Generalized estimating equations
HIV: Human immunodeficiency virus
MDT: Maggot debridement therapy
SD: Standard deviation
TBSA: Total body surface area
TGF-β: Transforming growth factor-beta
