# Supplementary material for: Larval therapy vs conventional silver dressings for full-thickness burns: a randomized controlled trial
Source: BMC Med. 2023 Sep 19;21:361. doi: 10.1186/s12916-023-03063-7 (PMC10510148; doi:10.1186/s12916-023-03063-7)
Supplement: Supplementary file 1 — Additional file 1. Sample size calculation. [file 12916_2023_3063_MOESM1_ESM.docx]

**Random assignment, allocation concealment, and calculation of sample size.**

This open randomized controlled trial was carried out at Shahid Motahari Burns Hospital, Tehran, Iran from November 2018 to May 2020. Participants were 31 cases with at least one full-thickness (grade-III based on ICD-10 classifications) burn that referred to Shahid Motahari Burns Hospital related to Iran University of Medical Sciences, Tehran, Iran. The flow of participants is shown in Figure 1. The patients were randomly assigned to either the Larvae or the Conventional groups by block randomization. A block size of four was used for randomization, and the sequences were calculated by Random allocation software v. 2.0. The research coordinator had exclusive access to the numbered, sealed, opaque envelopes that indicated each patient’s randomized treatment assignment. As an open label study, the patients, care-providers, and outcomes assessors were all informed to the treatment assignments. Indeed, the nature of the maggot therapy would not allow the study to be done in blinded manner. The sample size was calculated based on the time to debridement (primary outcome) in the Larvae group versus the Conventional group. We assumed the anticipated effect size of f = 1.05 (mean (SD)=10 (3) and 13.7(4.3) day Larvae group and Conventional group, respectively, type I error of 0.05 and test power of 90% based on Muangman P et al.’s study ([23](#_ENREF_23)). A total sample size of n = 34 was calculated with G*Power 3.1 (University of Kiel, Germany) using Two-sample independent t-test. However, after all participants completed at least 6 days of follow-up, an error was found in the original sample size calculation, and the planned 31 participants actually provided a power of 0.88 to detect a mean difference of 3.7 days. The statistic details of the sample size calculation are as follows.

G*Power:

calculation of sample size:

**Analysis:** A priori: Compute required sample size

**Input:** Tail(s) = One

Effect size d = 1.05

α err prob = 0.05

Power (1-β err prob) = 0.90

Allocation ratio N2/N1 = 1

**Output:** Noncentrality parameter δ = 3.0612497

Critical t = 1.6938887

Df = 32

Sample size group 1 = 17

Sample size group 2 = 17

Total sample size = 34

Actual power = 0.9116000

calculation of Post hoc power:

**t tests -** Means: Difference between two independent means (two groups)

**Analysis:** Post hoc: Compute achieved power

**Input:** Tail(s) = One

Effect size d = 1.05

α err prob = 0.05

Sample size group 1 = 15

Sample size group 2 = 16

**Output:** Noncentrality parameter δ = 2.9215550

Critical t = 1.6991270

Df = 29

Power (1-β err prob) = 0.8863750
