# Supplementary material for: Larval therapy vs conventional silver dressings for full-thickness burns: a randomized controlled trial
Source: BMC Med. 2023 Sep 19;21:361. doi: 10.1186/s12916-023-03063-7 (PMC10510148; doi:10.1186/s12916-023-03063-7)
Supplement: Supplementary file 2 — Additional file 2. Graphic presentations of participants received Larvae (cases 1-15) or conventional treatment (cases 16-31) from day 0 to day 6. [file 12916_2023_3063_MOESM2_ESM.docx]

| Additional file 1. Graphic presentations of participants received Larvae (cases 1-15) or conventional treatment (cases 16-31) from day 0 to day 6. | | | | |
| --- | --- | --- | --- | --- |
| **Larval therapy (cases1-15)** | | | | |
|  | Day 0 | Day 2 | Day 4 | Day 6 |
| Case 1 | 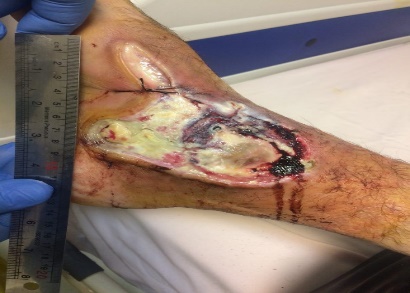 | 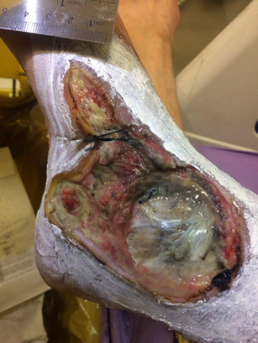 | 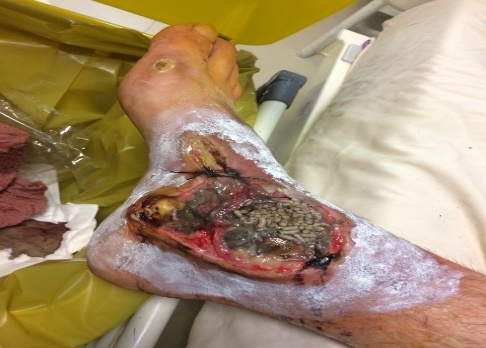 | 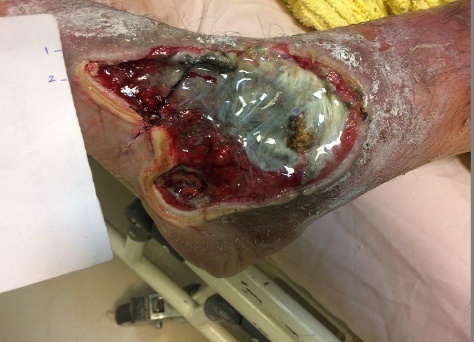 |
| Case 2 | 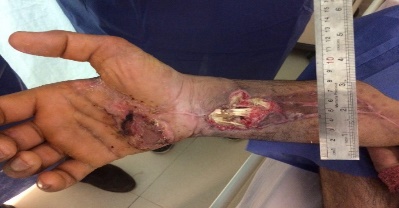 | 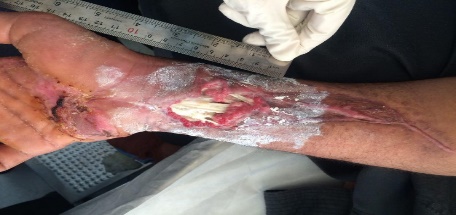 | 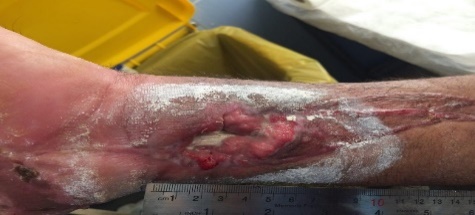 | 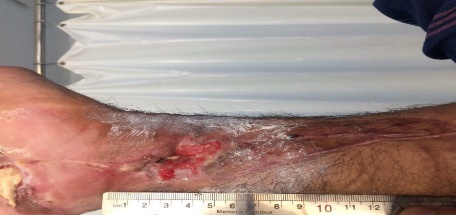 |
| Case 3 | 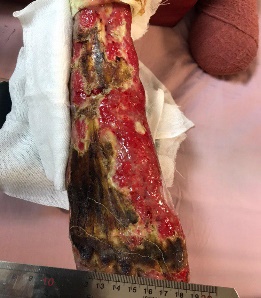 | 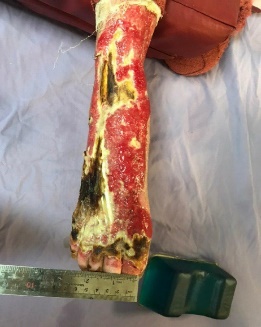 | 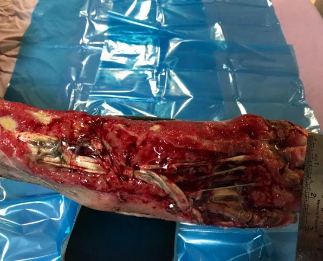 |  |
| Case 4 | 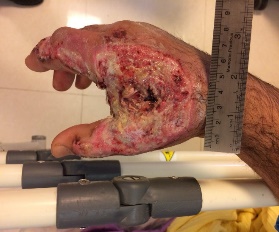 | 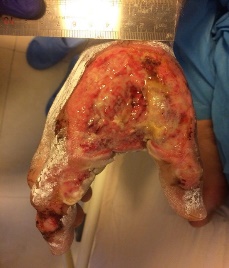 | 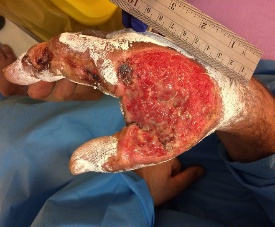 |  |
| Case 5 | 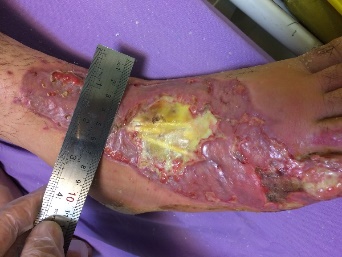 | 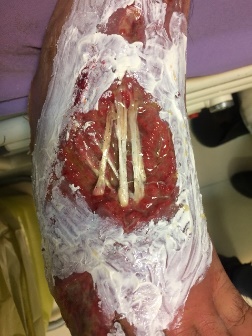 | 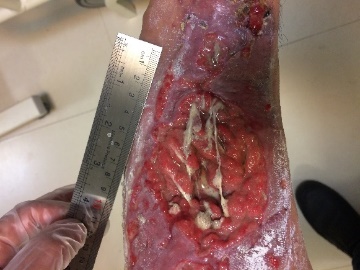 |  |
| Case 6 | 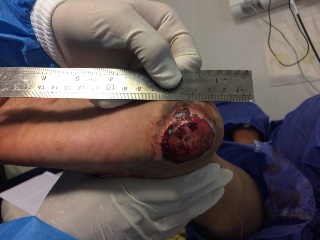 | 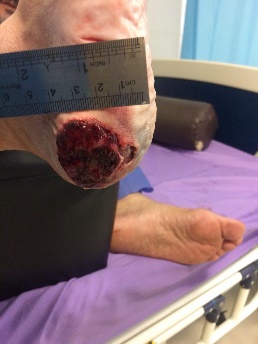 | 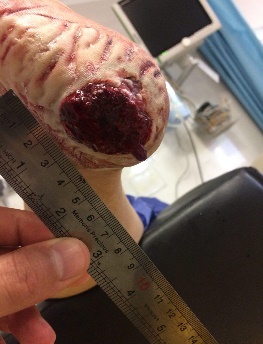 |  |
| Case 7 | 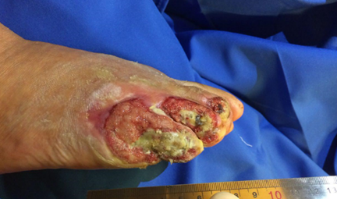 | 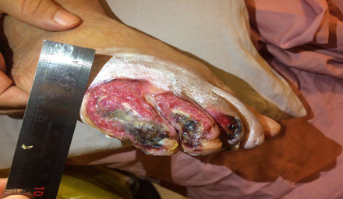 | 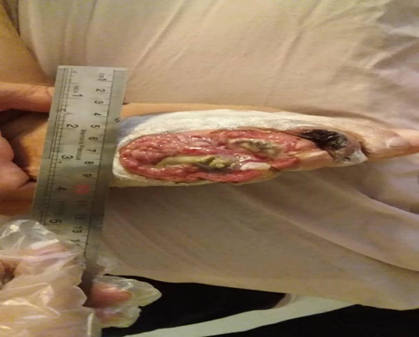 | 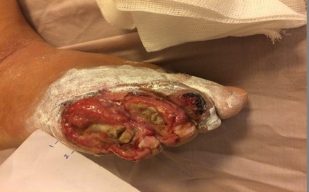 |
| Case 8 | 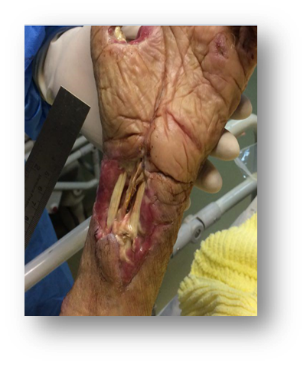 | 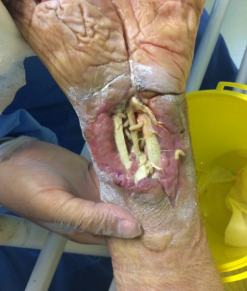 | 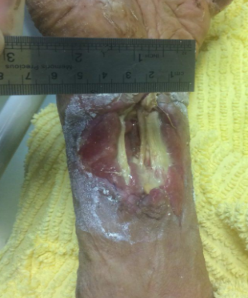 | 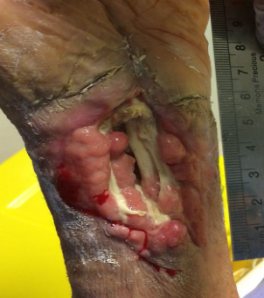 |
| Case 9 | 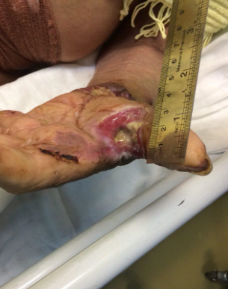 | 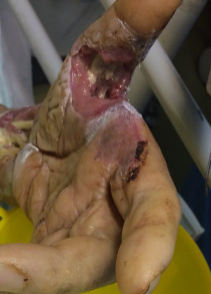 | 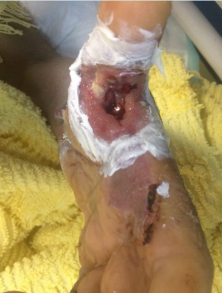 | 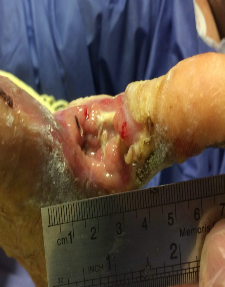 |
| Case 10 | 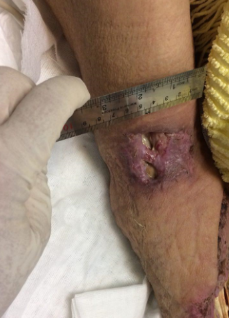 | 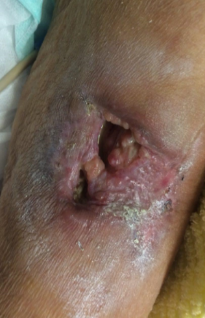 | 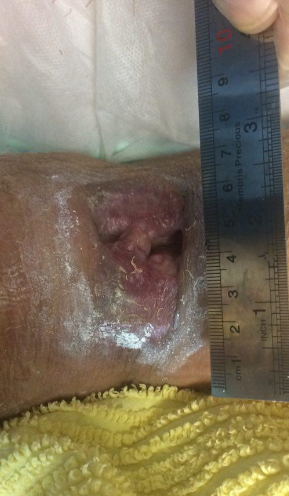 | 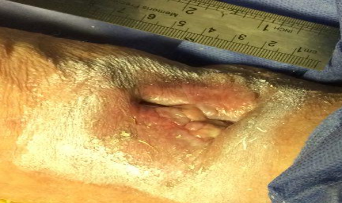 |
| Case 11 | 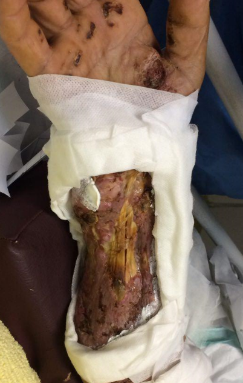 | 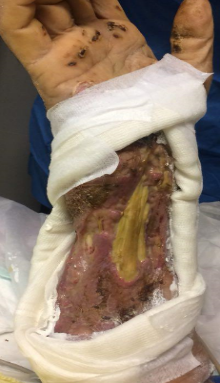 | 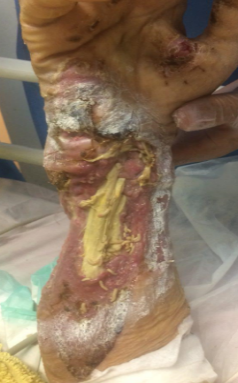 | 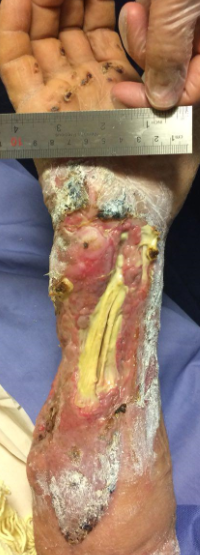 |
| Case 12 | 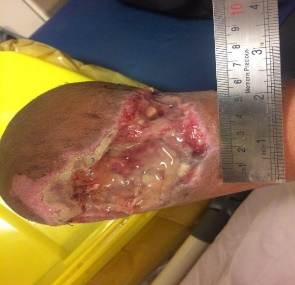 | 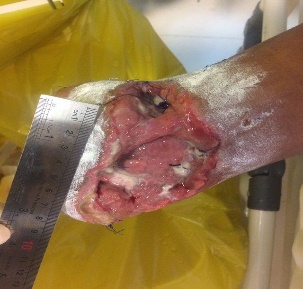 | 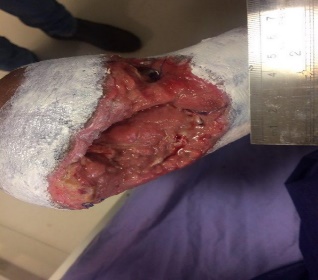 |  |
| Case 13 | 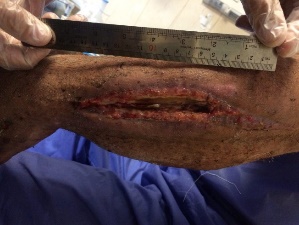 | 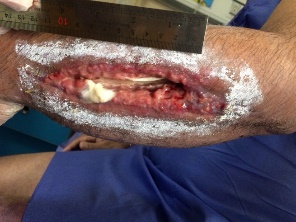 | 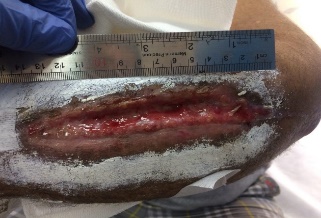 |  |
| Case 14 | 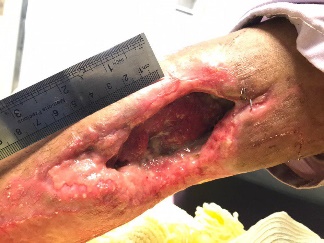 | 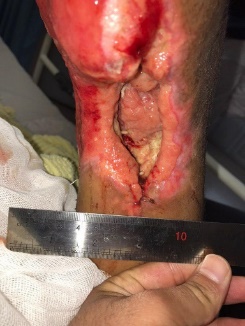 | 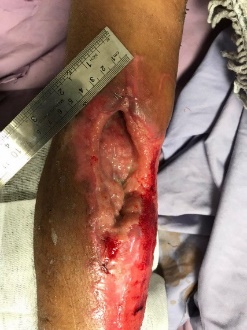 |  |
| Case 15 | 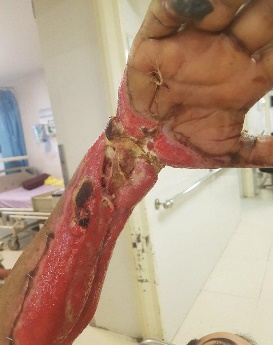 | 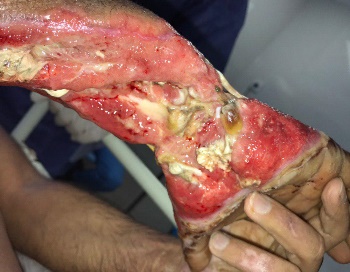 |  |  |
|  | **Conventional treatment (cases 16-31)** | | |  |
| Case 16 | 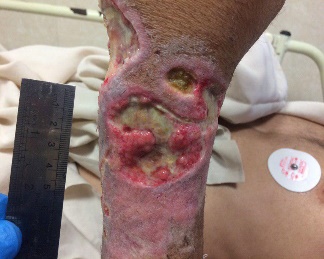 | 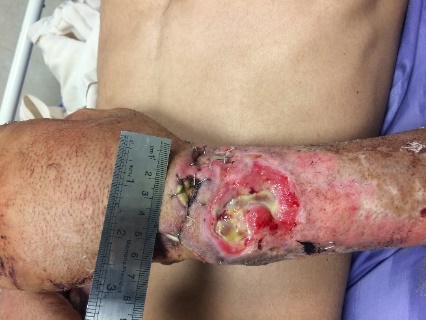 | 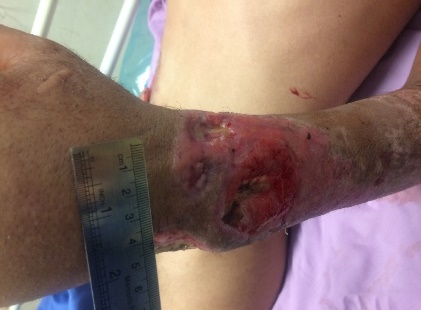 | 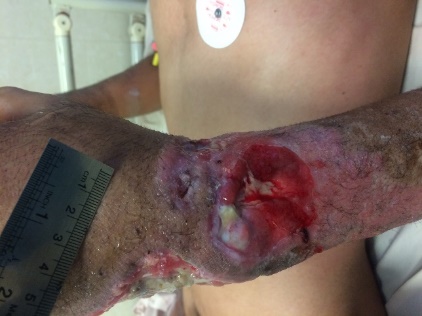 |
| Case 17 | 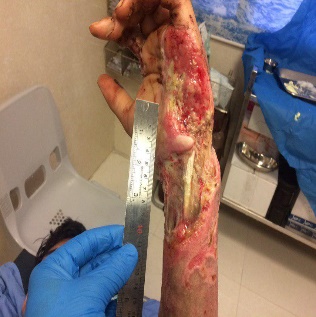 | 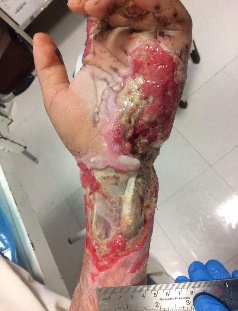 | 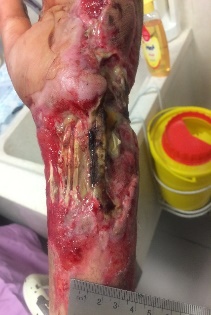 | 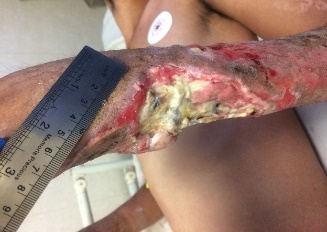 |
| Case 18 | 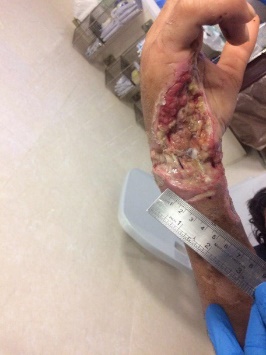 | 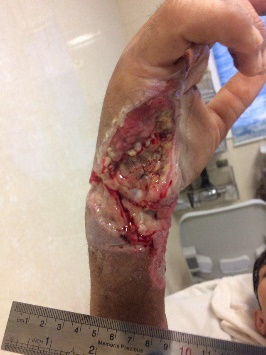 | 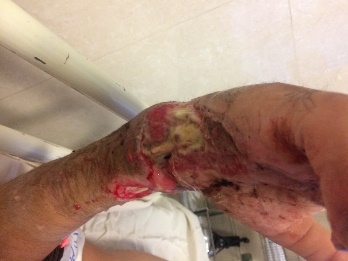 | 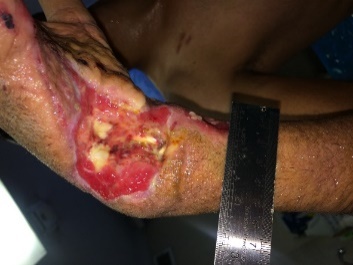 |
| Case 19 | 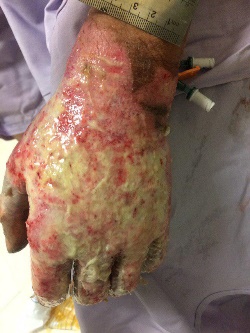 | 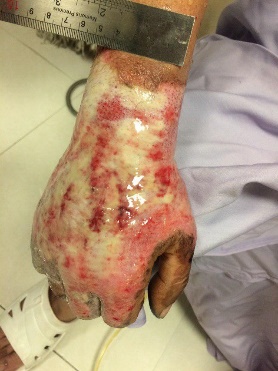 | 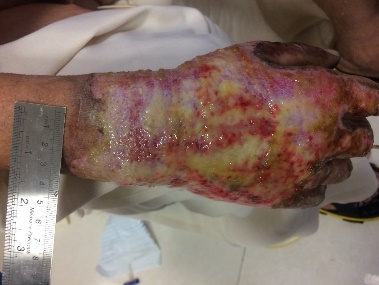 | 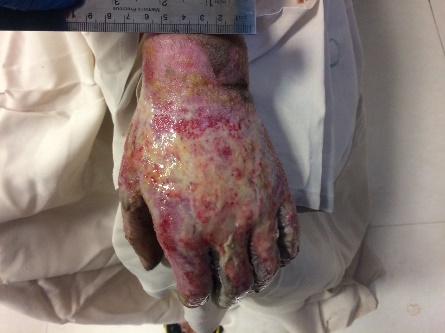 |
| Case 20 | 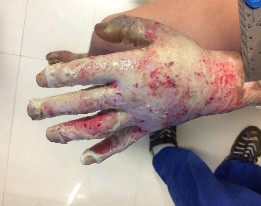 | 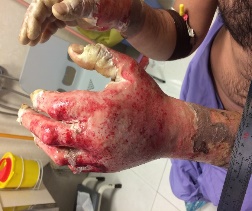 | 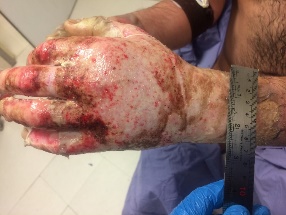 | 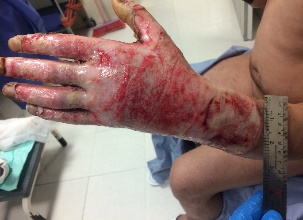 |
| Case 21 | 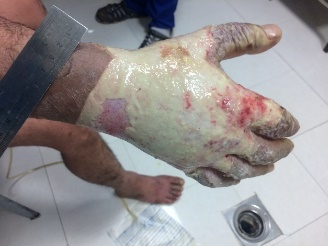 | 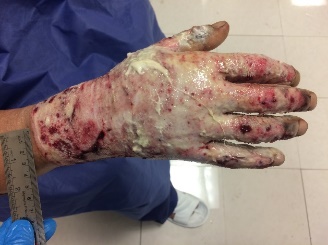 | 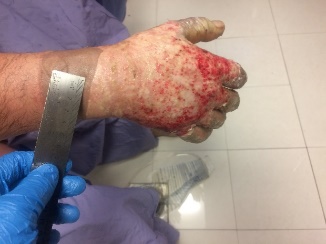 | 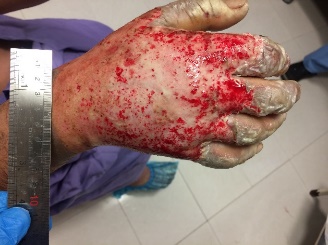 |
| Case 22 | 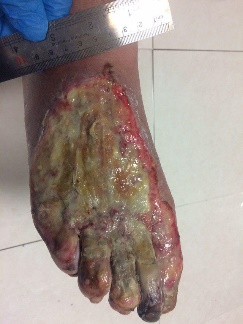 | 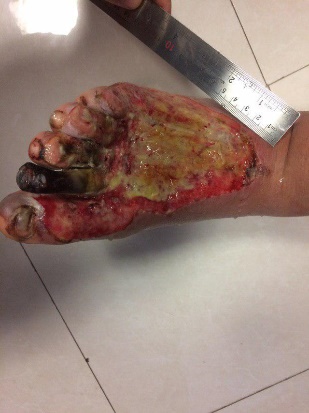 | 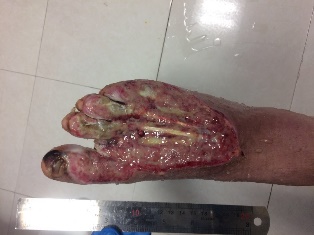 | 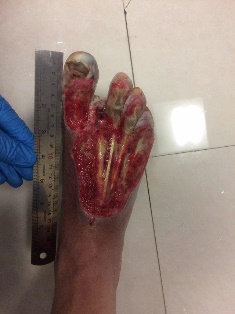 |
| Case 23 | 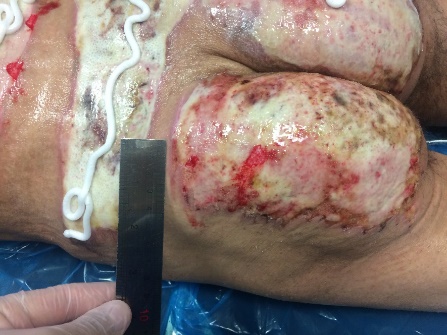 | 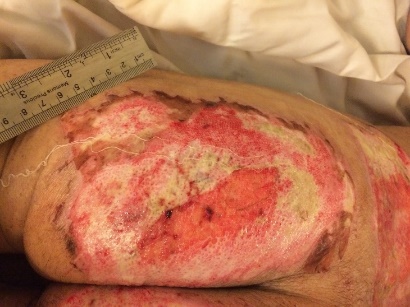 | 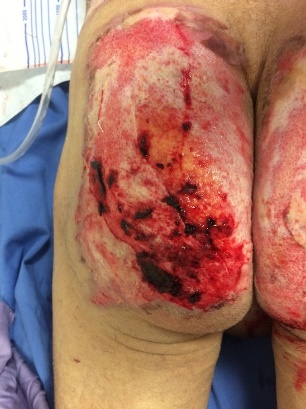 |  |
| Case 24 | 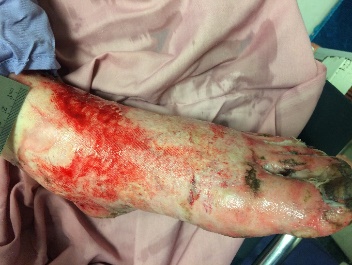 | 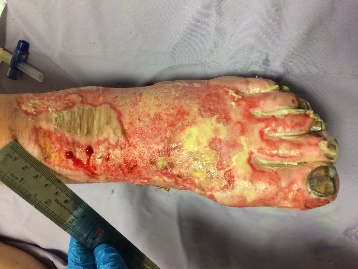 | 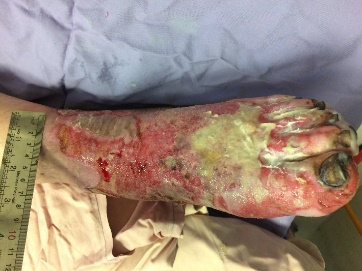 | 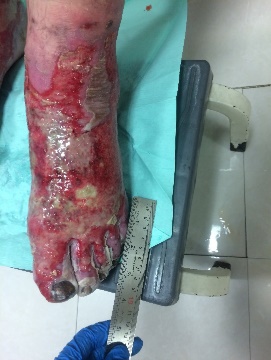 |
| Case 25 | 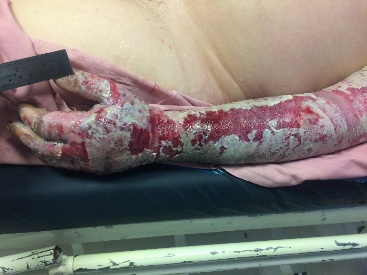 | 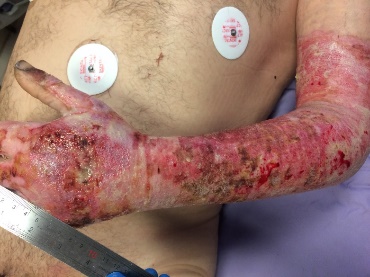 | 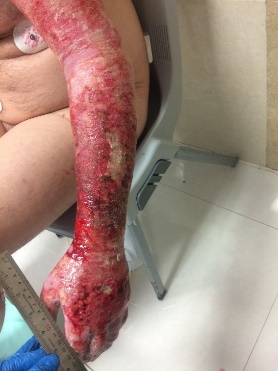 |  |
| Case 26 | 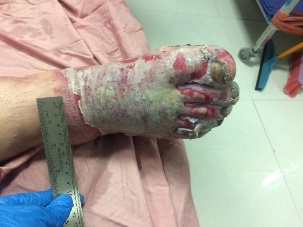 | 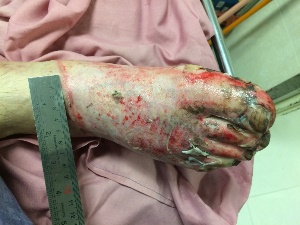 | 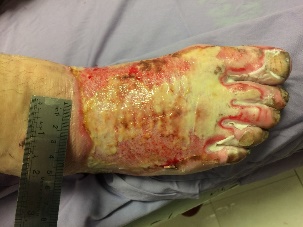 | 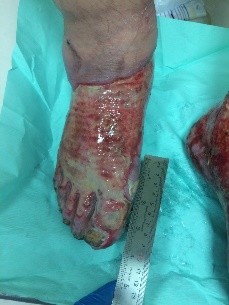 |
| Case 27 | 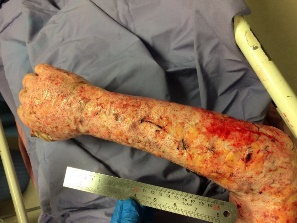 | 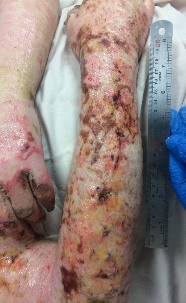 | 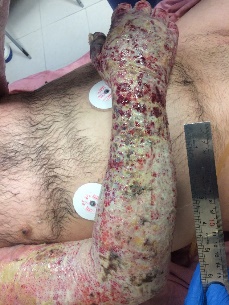 | 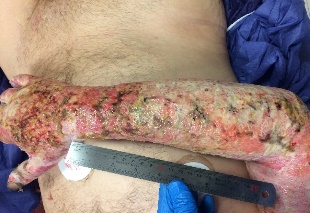 |
| Case 28 | 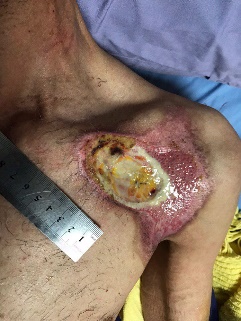 | 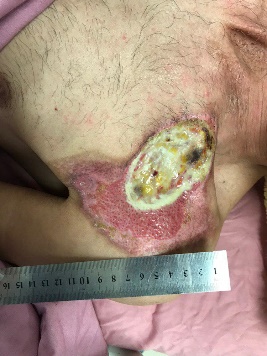 | 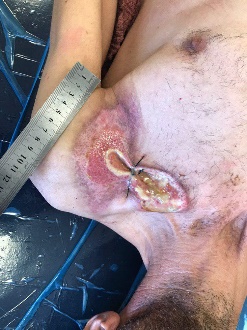 |  |
| Case 30 |  |  |  |  |
| Case 31 |  |  |  |  |
