# Supplementary material for: Larval therapy vs conventional silver dressings for full-thickness burns: a randomized controlled trial
Source: BMC Med. 2023 Sep 19;21:361. doi: 10.1186/s12916-023-03063-7 (PMC10510148; doi:10.1186/s12916-023-03063-7)
Supplement: Supplementary file 3 — Additional file 3. (Study protocol). [file 12916_2023_3063_MOESM3_ESM.docx]

**Study Protocol**

**Patients and randomization**

This open randomized controlled trial was carried out at Shahid Motahari Burns Hospital, Tehran, Iran from November 2018 to May 2020. Participants were 31 cases with at least one full-thickness (grade-III based on ICD-10 classifications) burn that referred to Shahid Motahari Burns Hospital related to Iran University of Medical Sciences, Tehran, Iran. The flow of participants is shown in Figure 1. Patients were randomized to receive either loose larvae or conventional treatment. All the cases in larvae group were informed about the methodology and study purposes and enrolled as volunteers, with a signed consent letter. Patients who had phobia or other serious per-exiting conditions including the presence of gangrene, severe pain, refractory to treatment, immunocompromised patients (e.g. HIV/AIDS), and those who have been receiving steroids and anti-coagulants that might affect the results were excluded from the study. Furthermore, probable bleeding because of larvae therapy and causing slough burns were also considered as exclusion criteria. All the procedures were in accordance to the Helsinki Declaration regarding human research, and were reviewed and approved by the Medical Ethics Board of Tehran University of Medical Sciences, Tehran, Iran (with code no. IR.TUMS.VCR.REC.1396.4691) ([23](#_ENREF_23)). This study has also been registered in Iranian registry of clinical trials and received a clinical trial code (IRCT ID: IRCT20170531034272N2).

37 patients excluded

- Other grades (I, II or IV) (n=20)
- Phobia (n=7)
- No consent (n=5)
- Pre-existing conditions (5)

Patients screened for eligibility (n=73)

Eligible patients (n=36)

Participants excluded

- 3 patients were excluded because of no collaboration.
- 2 patients were excluded because of Larvae phobia

Participants randomized (n=30+1)

Allocated to Larval therapy. (n=15)

Allocated to conventional treatment (n=16)

Discontinued intervention (non-discontinued)

Discontinued intervention (no patient)

Analyzed for ITT (15)

Analyzed for ITT (16)

Figure 1. Flow of participants enrolled in this study. The present study was conducted based on CONSORT guidelines for reporting clinical trials.

**Interventions**

As mentioned earlier, eligible participants were those who had full-thickness (grade-III based on ICD-10 classifications version 2019) burns based on the pathological examinations and expert opinions irrespective of the size and necrosis rate of wounds. Larvae group and controls were 15 and 16 patients with full-thickness (grade-III) burns, respectively. In the intervention group, each patient has received loose larvae (5-10 larvae/cm^2^ of burn area as described elsewhere for diabetic ulcers) for 3-4 times, with two days interval (day 0, day 2, day 4, and day 6) ([24](#_ENREF_24), [25](#_ENREF_25)). On the other hand, control group received the conventional regimen for full-thickness burns (sharp debridement, silver dressings, antibiotic therapy, offloading). Of note, in the conventional treatment group, burn dressings were replaced every day by expert nurses.

**Data collection and outcome measurements**

Demographics of patients including age, sex and baseline data on burn mechanisms (exposure source), site of burning, necrotic and granulation tissues were collected at admission to hospital and summarized in Table 1. Furthermore, related data on burns after the application of treatments was measured every 48 hrs (during the replacement of dressings) under the observation doctors specialized in dermatology and burning. To examine the therapeutic effects of larvae in comparison to conventional treatment, the surface areas of granulation and necrotic tissues were photographed at days 0, 2, 4, and 6 and analyzed by Image J Software. To minimize errors, all these calculations were repeated three times and optimized with expert opinion. Of special note, since the size of wounds were different in two groups, the results were adjusted to the would size to minimize the interpretation bias and to detect more precise statistically significant differences.

The primary outcome in this study was considered as time to debridement that was defined as the time in which burns were cosmetically clean and participants were ready to receive transplantation. Digital photographs were taken during every two days, when old larvae dressings were replaced with new ones, and are presented for each patients in supplementary file, with a representation for each group in figure 2. The secondary outcome was considered as the time to healing that was defined as the duration from the admission to the complete healing post-transplantation based on the experts' opinion.
